# Research Progress on Na_3_V_2_(PO_4_)_3_ Cathode Material of Sodium Ion Battery

**DOI:** 10.3389/fchem.2020.00635

**Published:** 2020-07-24

**Authors:** Xianguang Zeng, Jing Peng, Yi Guo, Huafeng Zhu, Xi Huang

**Affiliations:** ^1^Institute of Material and Chemical Engineering, Sichuan University of Science and Engineering, Zigong, China; ^2^Material Corrosion and Protection Key Laboratory of Sichuan Province, Zigong, China; ^3^Zigong Langxingda Technology Co., Ltd., Zigong, China

**Keywords:** Na_3_V_2_(PO_4_)_3_, cathode material, research progress, sodium ion batteries, prepare and modification

## Abstract

Sodium ion batteries (SIBs) are one of the most potential alternative rechargeable batteries because of their low cost, high energy density, high thermal stability, and good structure stability. The cathode materials play a crucial role in the cycling life and safety of SIBs. Among reported cathode candidates, Na_3_V_2_(PO_4_)_3_ (NVP), a representative electrode material for sodium super ion conductor, has good application prospects due to its good structural stability, high ion conductivity and high platform voltage (~3.4 V). However, its practical applications are still restricted by comparatively low electronic conductivity. In this review, recent progresses of Na_3_V_2_(PO_4_)_3_ are well summarized and discussed, including preparation and modification methods, electrochemical properties. Meanwhile, the future research and further development of Na_3_V_2_(PO_4_)_3_ cathode are also discussed.

## Introduction

**Figure 1:**
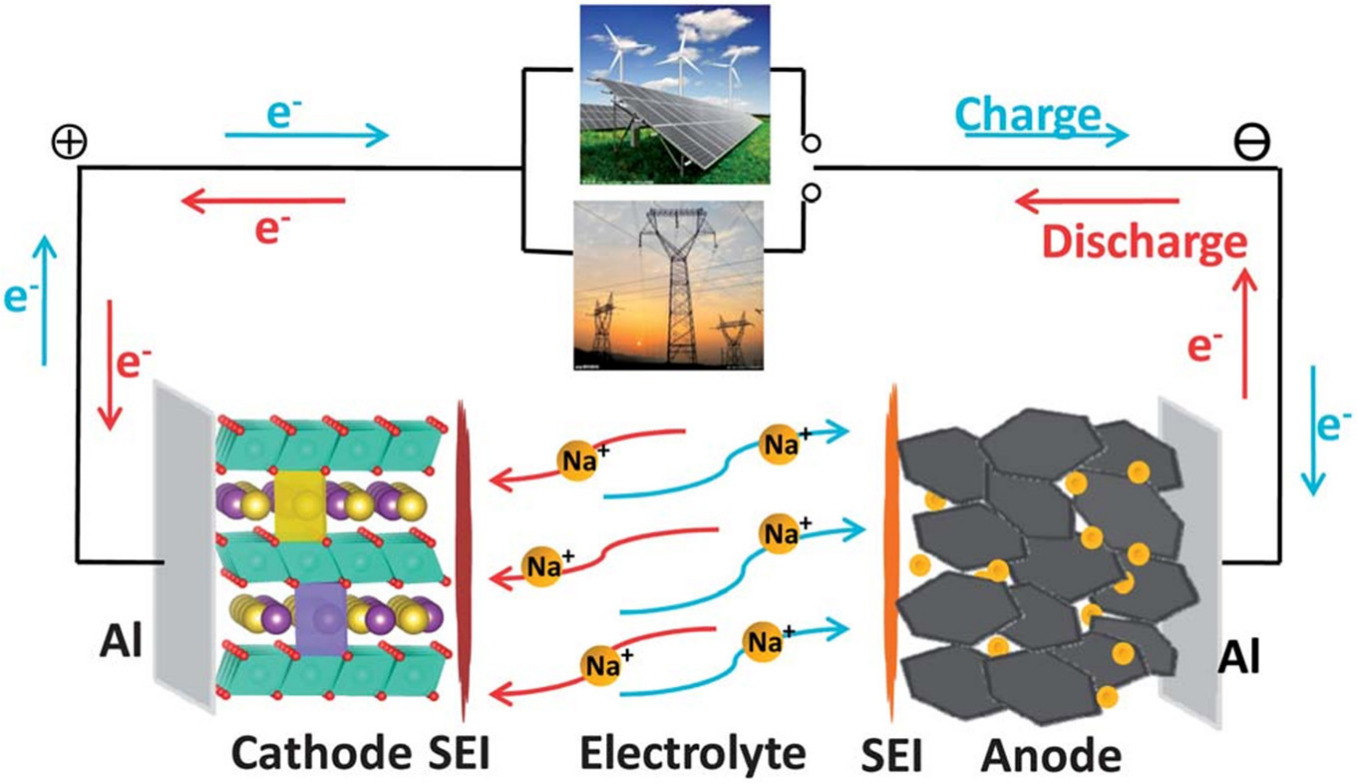
Principe diagram of sodium ion battery work.

Lithium-ion battery (LIB), a kind of rechargeable battery, has been designed and modified to power portable electronic equipment, electric vehicle and even energy storage power station because of its high energy density, high voltage, and environmentally friendly (Goodenough and Park, 2013; Song et al., 2014a; Han et al., 2018; Yi T. F. et al., 2018; Fang R. et al., 2019; Li et al., 2019; Fang et al., 2020; Nie et al., 2020a,b; Wang et al., 2020). However, large-scale energy storage applications of LIBs could be hindered by the high cost of lithium minerals (Jiang et al., 2020; Yi et al., 2020). Hence, finding an alternative and sustainable electrochemical battery is necessary (Yang et al., 2011; Yabuuchi et al., 2014; Liu et al., 2018; Mao et al., 2018). Among the optional energy storage systems, sodium ion batteries (SIBs) have strongly caught researcher's attentions on account of abundant sodium resources, high energy storage capacities and high electrochemical activity (Komaba et al., 2011; Lee et al., 2011; Ponrouch et al., 2012; Tepavcevic et al., 2012; Chang et al., 2013; Slater et al., 2013; Farbod et al., 2014; Li W. et al., 2014; Xie et al., 2014; Zhong et al., 2016; Zhu et al., 2016; Li and Zhou, 2018; Nayak et al., 2018; Song et al., 2018; Vaalma et al., 2018). Though SIBs have similar structure with lithium ion batteries (Figure 1) (Li et al., 2013; Palomares et al., 2013; Pan et al., 2013; Kundu et al., 2015; Hwang et al., 2017; Zhao, 2019), Na ion has lager radium than lithium ion and easily coordinate in crystalline materials. Therefore, exploring appropriate host materials or other high energy density cathode materials are necessary for the developing of SIBs.

Suitable cathode materials should allow rapid Na-ion transport but also maintain structural stability and against the structural distortion/volume change in the process of Na ion extraction/insertion (Qi et al., 2015; Fang et al., 2016; Lee et al., 2016; Liu et al., 2016). The cathode materials of SIBs mainly include lamellar materials (Komaba et al., 2010; Kim et al., 2011, 2012; Liao et al., 2013; Tu et al., 2017; Xiao Y. et al., 2018), polyanionic materials (Ong et al., 2011; Fang et al., 2017) and polymer materials (Hwang et al., 2017; Deng et al., 2018). Among them, layered Na_x_MnO_2_ (Caballero et al., 2002) and Na_x_CoO_2_ (Samin et al., 2012), as well as phosphates-based NaMPO_4_ (M = Fe, Co, Ni, Mn) (Oh et al., 2012; Zhu et al., 2012; Hasa et al., 2014) have been deeply studied. Especially, sodium super ion conductor (NASICON) structured composite, such as Na_3_V_2_(PO_4_)_3_ (NVP) is likely to be the best candidate, because it has high theoretical energy storage capacities (117.6 mAh g^−1^), and rich Na-ion transport channels resulting from its open three-dimensional (3D) framework. However, the NVP has low electronic conductivity, which is not good for the migration of electrons. In order to solve this issue, many efforts have been done. For example, preparing nano-scaled Na_3_V_2_(PO_4_)_3_ to reduce the diffusion path of Na^+^ and accelerate its transportation. In addition, coating NVP with conductive carbon/polymer materials, or modifying Na_3_V_2_(PO_4_)_3_ with heterogeneous elements are also valid means to improve the electric conductivity of NVP.

In this minireview, the recent progresses of NVP are well summarized and discussed, including preparation methods, modification means (nanostructure, carbon coating, element doping) and their effects on the electrochemical property of NVP cathode.

## Structure of Na_3_V_2_(PO_4_)_3_

**Figure 2:**
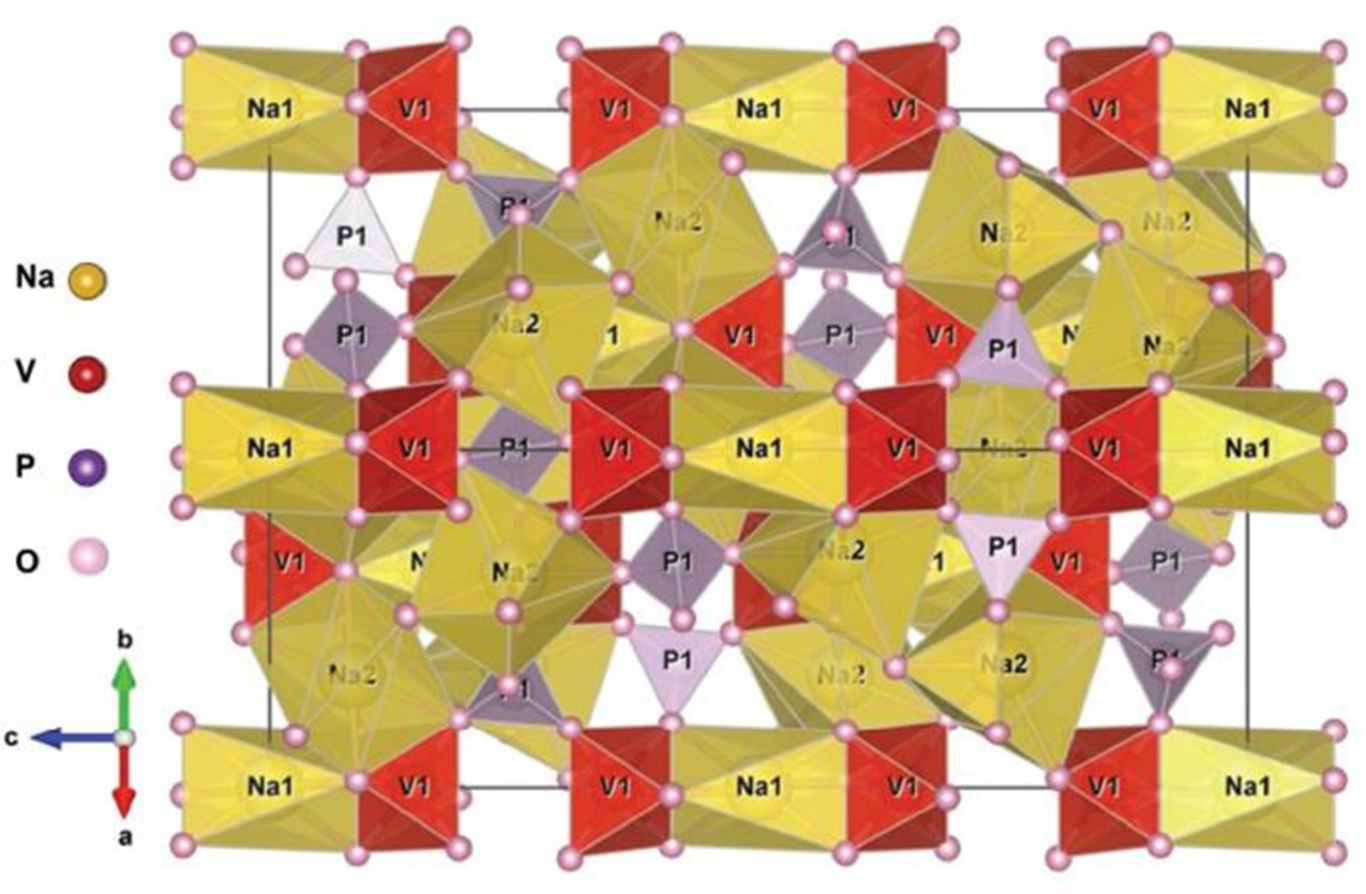
Structure scheme of Na_3_V_2_(PO_4_)_3_.

Served as a greatly hopeful cathode material of SIBs, NVP crystallizes have trigonal system and belong to R-3c space group. As shown in Figure 2, VO_6_ octahedra and PO_4_ tetrahedra interlink mutually to construct a 3D [V_2_(PO_4_)_3_] frame via sharing corners (Kabbour et al., 2011; Kang et al., 2012; Shen et al., 2015a; Lavela et al., 2018), in which sodium ion occupies two different positions of Na(1) and Na(2), respectively. The desorption process of sodium ion relates to the transformation from Na_3_V_2_(PO_4_)_3_ to NaV_2_(PO_4_)_3_, it is generally considered that all the outer sodium ions come from the Na(2) position while the Na(1) position is unchanged. Though two sodium are stripped, the frame of NVP can still be maintained due to the strong covalent effect of (PO_4_)^3−^, leading to a high capacity of 117.6 mAh g^−1^ (Zheng et al., 2018).

## Synthetic Methods of Na_3_V_2_(PO_4_)_3_

Synthetic methods play an important role in controlling the morphology and particle size of electrode materials, which will further affect their electrochemical performance. Several approaches to synthesize Na_3_V_2_(PO_4_)_3_ electrode materials for SIBs, such as sol-gel method (Lim et al., 2012; Pivko et al., 2012; Li et al., 2015a; Wang S. Y. et al., 2015; Klee et al., 2016a; Song et al., 2016), hydrothermal method (Li H. et al., 2014; Nie et al., 2014; Ren et al., 2016), solid-state method (Gopalakrishnan and Rangan, 1992; Zatovsk, 2010; Du et al., 2013; Zhu et al., 2014; Klee et al., 2016b), and electrospinning method (Kajiyama et al., 2014; Li et al., 2015b) are summarized as follow.

### Sol-Gel Method

Sol-gel method is the most common method to synthesize Na_3_V_2_(PO_4_)_3_, which converts colloidal suspension (sol) into a whole 3D network (gel) with submicron scale pores. Compared with other approaches, Sol-gel method has lower operating temperature and the preparation process is easy to control. However, it usually needs high cost and complex preparation routes. Hence, it does not always meet the industrial demands (Zhou et al., 2009; Rui et al., 2014; Wang D. X. et al., 2015).

**Figure 3:**
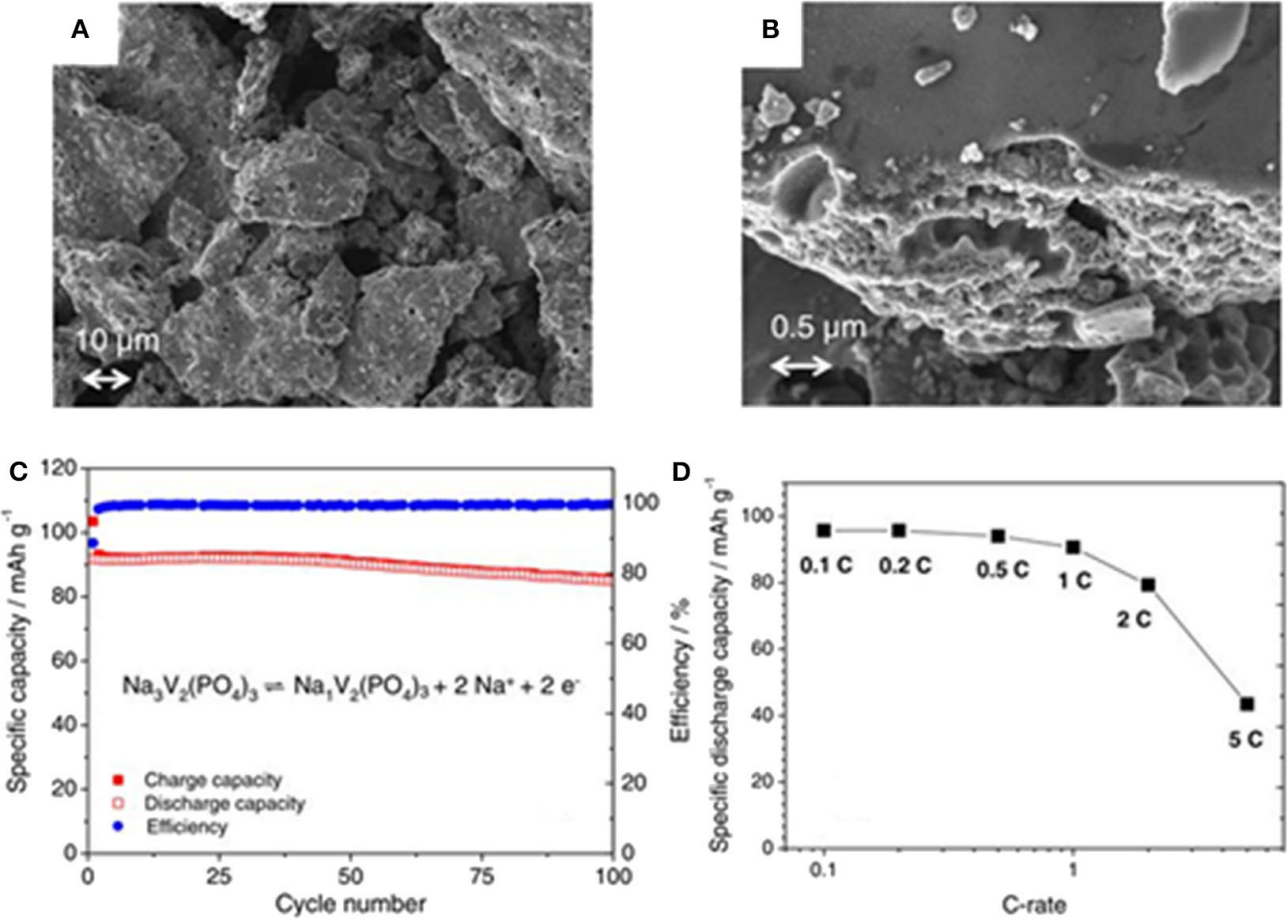
**(A,B)** The SEM images of NVP/C, **(C)** constant-current charge/discharge of NVP-based electrodes material at 1 C, **(D)** C-rate test of Na_3_V_2_(PO_4_)_3_-based electrodes.

Wang et al. (2019) have successfully prepared a Na_3_V_2_(PO_4_)_3_ cathode material by a typical sol-gel method using citric acid as complexant. The as-obtained Na_3_V_2_(PO_4_)_3_ sample exhibits a high initial discharge capacity of 107 mAh g^−1^ and high reversible capacity (97.1 mAh g^−1^) after 150 cycles at 0.2 C. A Na_3_V_2_(PO_4_)_3_ cathode material coated by carbon has been prepared by standard sol-gel method, which shows particles size range from 10 to 20 μm (Figures 3A,B) (Böckenfeld and Balducci, 2014). Results from galvanostatic intermittent titration technique (GITT) and cyclic voltammetry (CV) test reveal the apparent diffusion coefficient of sodium ions in the rhombohedral NVP. It ranges from 6 × 10^−13^ to 2 × 10^−15^ cm^2^ s^−1^, following an alike tendency that lithium ions behave in monoclinic Li_3_V_2_(PO_4_)_3_, indicating that the potential in ion extraction/insertion is minimum. Figure 3C shows the cycling performance over 100 cycles at 1 C. As described, the cathode material displays a discharge capacity of 92 mAh g^−1^ at the first cycle and 85 mAh g^−1^ at the 100th cycle, corresponding to a capacity retention rate of 92%. It also presents good rate behavior (Figure 3D). When cycled from 0.1 to 0.5 C (corresponding to currents of 11.9 to 59.5 mAh g^−1^), and values in the order of 95 mAh g ^−1^.

### Hydrothermal Method

Hydrothermal method is a liquid chemical synthesis approach that can guarantee a homogeneous particle size distribution and high purity. Therefore, the hydrothermal method becomes one of the most common methods to synthesize the electrode materials. However, it should be noted that the hydrothermal method is not easily detected because the reactions are carried out in a kettle, thus making the process difficult to monitor (Liu et al., 2004; Gao et al., 2013).

**Figure 4:**
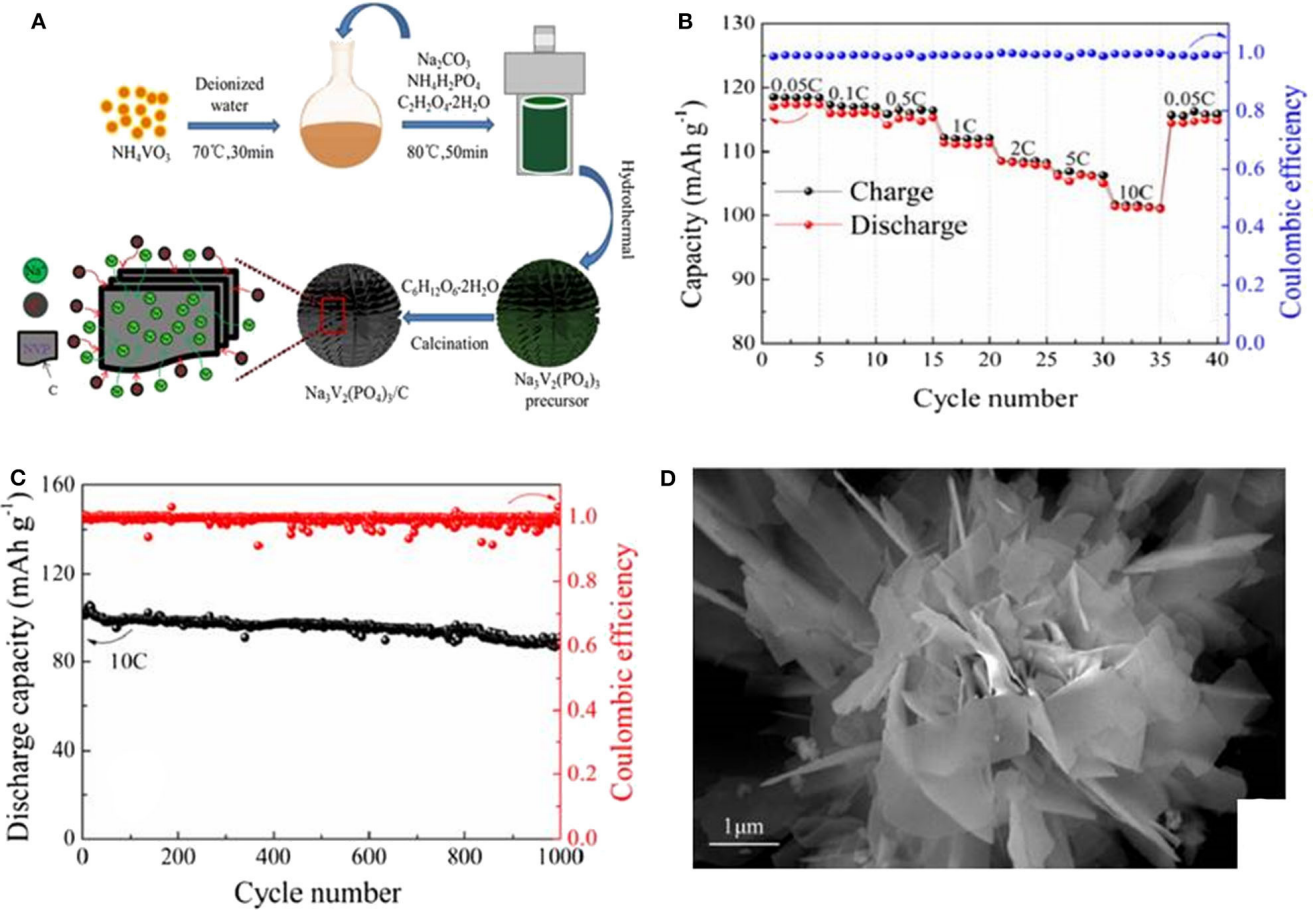
**(A)** Schematic illustration ofthe fabrication of NVP/C materials, **(B)** rate performance of NVP/C, **(C)** cycle performance of the NVP/C cycled at 10 C for 1,000 cycles, **(D)** SEM image of the NVP/C at 10 C for 1,000 cycles.

Wang (2019) have obtained Na_3_V_2_(PO_4_)_3_ by one-step hydrothermal method. According to their report, the as-synthesized Na_3_V_2_(PO_4_)_3_ has a discharge specific capacity of 89.3 mAh g^−1^ at the first cycle, and after 30 cycles, the capacity increases to 91 mAh g^−1^, signifying a good cycling performance. Ruan (Ruan et al., 2017) also successfully synthesizes a new kind of chrysanthemum structure Na_3_V_2_(PO_4_)_3_ and carbon composite (NVP/C) cathode material, and corresponding fabrication process is presented in Figure 4A. During the sodium ion diffusion process, scattering nanosheets in chrysanthemum petals are good for reducing energy consumption, while the carbon-coated layer can obviously boost the entirety electrochemical behavior. As a result, the sample shows an excellent electrochemical property because of its characteristic structure. It processes a premier discharge capacity of 117.4 mAh g^−1^ at 0.05 C and an ultra-high specific capacity of 101.3 mAh g^−1^ at 10 C (Figure 4B). Furthermore, it can maintain a high discharge capacity of 87.5 mAh g^−1^ after 1,000 cycles at 10 C, as displayed in Figure 4C. Figure 4D shows the scanning electron microscope(SEM) image of the NVP/C after cycling, it clearly exhibits that the chrysanthemum structure can still be maintained even after 1,000 cycles at 10 C. Therefore, the improved electrochemical property of NVP/C can be attributed to its excellent structure stability.

### Solid-State Method

Solid-state method, a traditional method of preparing electrode materials, is widely used in large-scale industrial application because of controllable reaction conditions, low cost and simple operation process. It should be noted that solid-state method still faces many challenges, such as anomalous morphologies and inhomogeneity of products.

**Figure 5:**
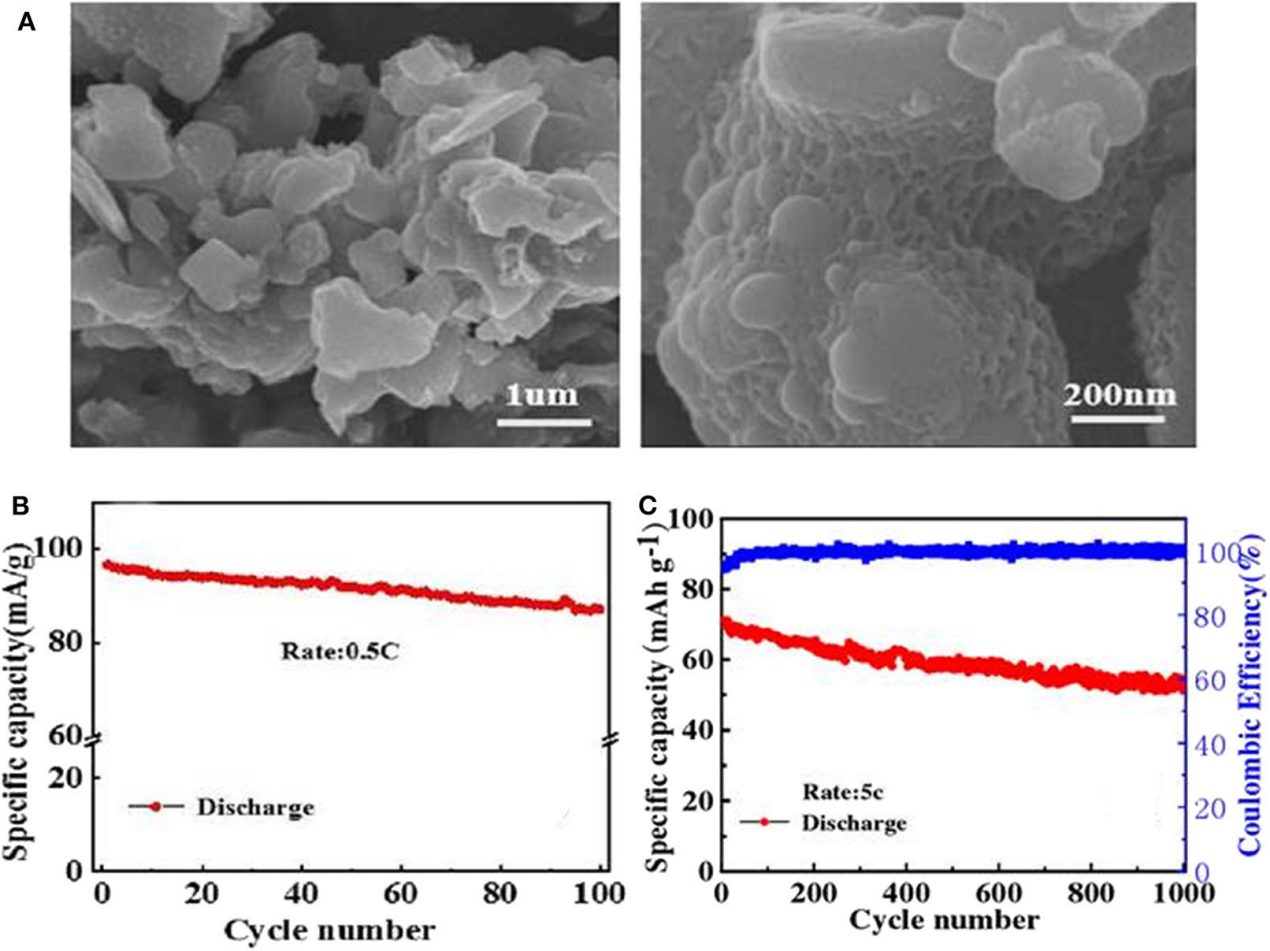
**(A)** SEM images of NVP/C, **(B)** cycle performance of the NVP/C, **(C)** cycle performance of the NVP/C cycled at a rate of 5 C for 1,000 cycles.

A carbon-coated Na_3_V_2_(PO_4_)_3_ cathode material (NVP/C) is prepared through the simple and easy-to-operate solid-state method (Zhu, 2019). The as-prepared NVP/C composite exhibits some porous network structure (Figure 5A), which are good for increasing specific surface area, promoting electrolyte infiltration, and facilitating transmissions of sodium ions. As a consequence, the electrochemical performances of NVP after modification are significantly enhanced. NVP/C cathode material shows an initial discharge capacity of 95.6 mAh g^−1^ at 0.5 C (Figure 5B), signifying a good cycling performance. When cycled at 5 C, it can still deliver a high capacity of 71.39 mAh g^−1^ and go through 1,000 cycles with a capacity retention of 72.3% (Figure 5C). Jian (2012) also reports a carbon-coated Na_3_V_2_(PO_4_)_3_ material made by one-step solid phase method, which manifests a discharge capacity 107.1 mAh g^−1^ at 0.1 C and obtains a lifespan over 80 cycles with a retention of 92.9%.

### Other Synthesis Methods

**Figure 6:**
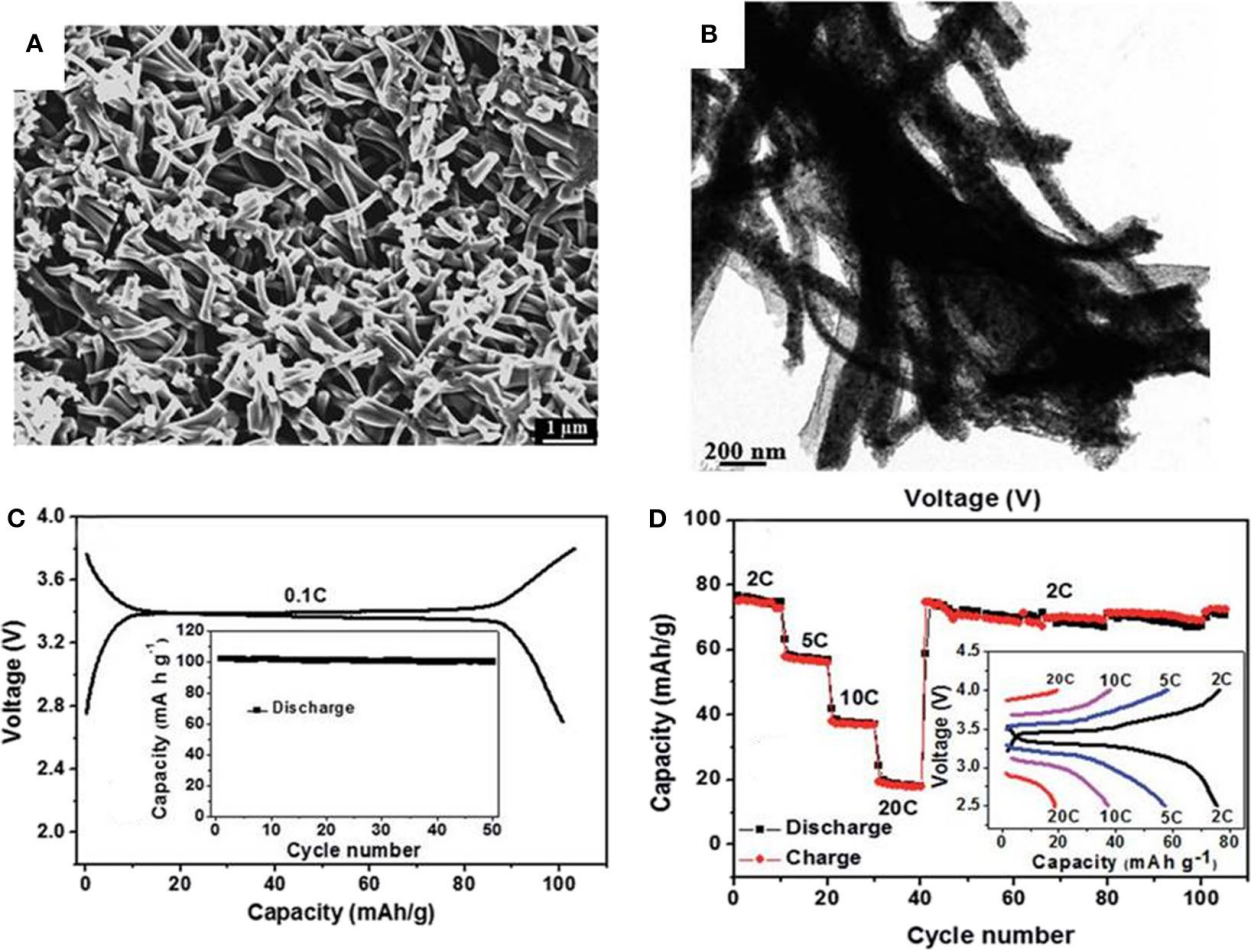
**(A)** SEM image of NVP/C, **(B)** low-magnification TEM image, **(C)** the first Charge-discharge curve at 0.1 C rate, **(D)** cycling performance of the NVP/C cathode at different current densities.

Except for the technologies mentioned above, electrospinning is a method to fabricate fiber structure electrode materials. Compared with other methods, electrospinning can satisfy the large-scale industrial preparation and able to prepare uniform materials. Liu et al. (2014) use 20–30 nm NVP nanoparticles and citric acid as reactants to prepared a framework Na_3_V_2_(PO_4_)_3_/carbon (NVP/C) composite material by a simple electrostatic spinning and subsequent carbonization strategy. Figure 6A shows the SEM image of the final composite, it is obvious that NVP/C nanofibers interweave each other to form a 3D network. Figure 6B shows the transmission electron microscopy(TEM) images of NVP/C, where NVP are coated uniformly by thin carbon layer and form composite fiber with diameters about 200 nm. Ascribing to the 3D crosslinked conductive network, the as-obtained cathode material exhibits high charge (discharge)capacity of 103(101) mAh g^−1^ at 0.1 C (Figure 6C) and manifests a stable discharge capacities of 77, 58, 39, and 20 mAh g^−1^ at 2, 5, 10, and 20 C, respectively (Figure 6C).

In summary, synthesis methods can obviously affect the structure and morphology of NVP cathode materials and further determine their electrochemical performance. Sol-gel method is widely used for obtaining electrode materials with homogeneous particle size. Hydrothermal treatment can guarantee high specific surface area and high purity. Solid-state method has the merits of low cost and simple operation process. Electrospinning is suitable to large-scale industrial preparation and able to prepare the uniform materials. Herein, the electrochemical performances of NVP prepared by different methods are compared and listed in Table 1.

**Table 1:** Electrochemical performances of NVP prepared by different methods.

**Synthetic methods**	**Rate/C**	**Cycles**	**Capacity/mAh g^**−1**^**
Sol-gel method (Kajiyama et al., 2014)	0.2	150	97
Sol-gel method (Li et al., 2015b)	1	100	85
Hydrothermal method (Wang D. X. et al., 2015)	0.2	30	91
Hydrothermal method (Wang et al., 2019)	10	1,000	87.5
Solid-state method (Böckenfeld and Balducci, 2014)	5	1,000	52
Solid-state method (Liu et al., 2004)	0.1	80	99.5

As shown, Na_3_V_2_(PO_4_)_3_ with excellent electrochemical property should have homogeneous particle size distribution and high specific surface area, which are convenient to the diffusion of Na^+^. In this respect, hydrothermal method behavior much better than other preparing technologies, however, considering the industrial application, this methods need to be improved or coordinated with other approaches.

## Modification Approaches of Na_3_V_2_(PO_4_)_3_

In other way, modification approaches including element doping (Aragón et al., 2015a,b; Fang et al., 2015; Shen et al., 2015b; Xu and Sun, 2016; Zhou W. D. et al., 2016; Chen L. F. et al., 2017; Zheng Q. et al., 2017; Li et al., 2018; Xiao H. et al., 2018; Zhao et al., 2018; Zhu et al., 2018; Fang J. Q. et al., 2019), and nanostructures (Huang et al., 2002; Li S. et al., 2014; Li et al., 2015c; Chu and Yue, 2016; Chen S. Q. et al., 2017; Wei et al., 2017; Zhang C. Z. et al., 2017) can also deeply influence the cycling life and rate performance of NVP.

### Element Doping

Adding heterogenous ions with larger ionic radius into the Na_3_V_2_(PO_4_)_3_ crystaline is an effective way to increase crystal volume, thereby expanding the tunnel for the diffusion of Na^+^. Furthermore, this method can also increase active sites. Hence, element doping is usually considered as a useful way to improve the performance of electrode materials.

**Figure 7:**
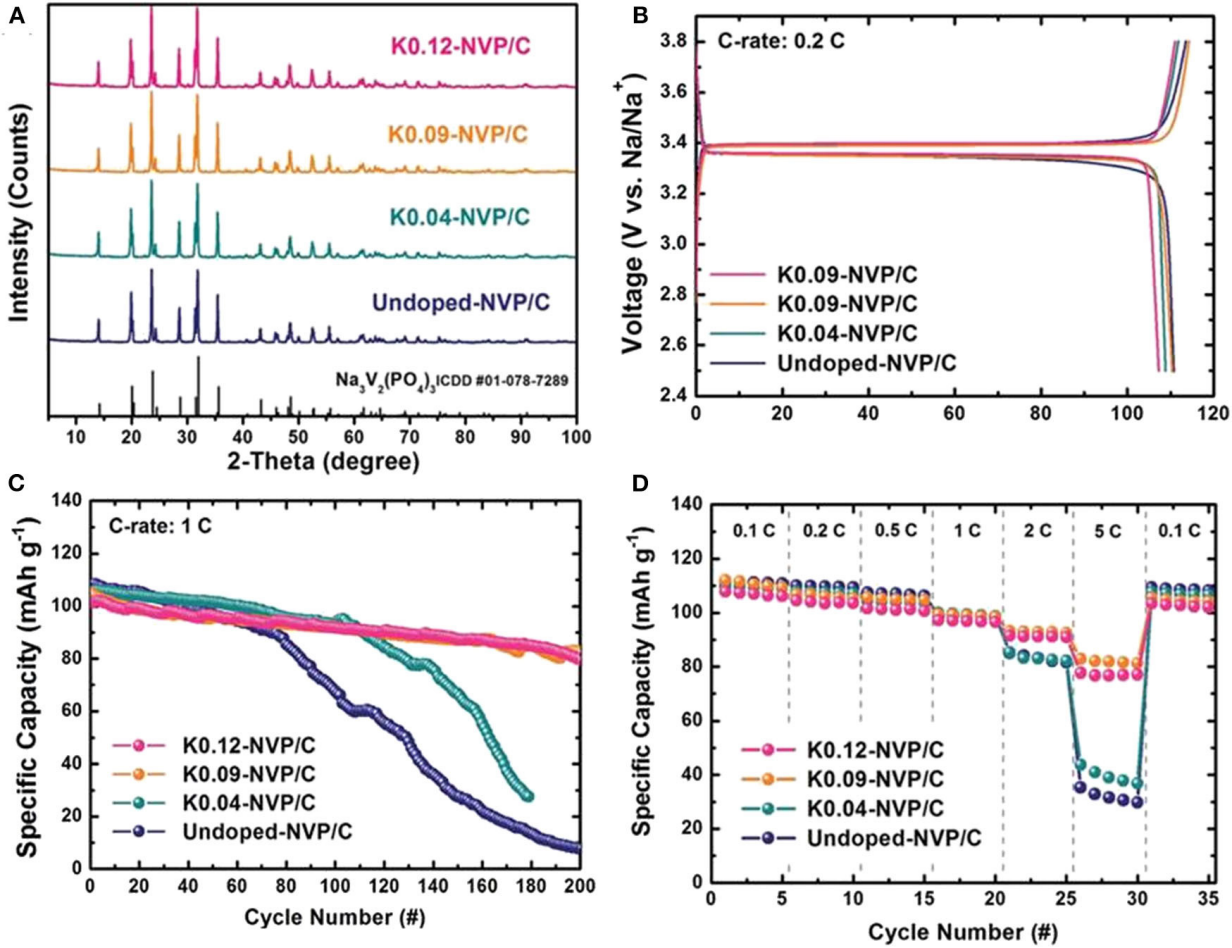
**(A)** XRD patterns of NVP/C doped with K^+^, **(B)** initial charge-discharge curve, **(C)** cycle property of NVP/C doped with K^+^, **(D)** rate capabilities of NVP/C doped with K^+^.

Lim et al. (2014) use K as heterogeneous element to enlarge the diffusion accesses of Na^+^ and maintain the NASICON framework during repeated cycling process, obtaining a significant improvement of electrochemical performance. The XRD patterns in Figure 7A reveal that the addition of K^+^ cannot change the crystal structure of NVP. Even there are no obvious difference in initial charge/discharge capacity between undoped NVP/C and doped NVP/C (Figure 7B), the cycling stability and rate performance of doped NVP/C cathode material with doping content of 0.09 and 0.12 increase significantly (Figures 7C,D).

Shen et al. (2015a) utilize B doped carbon to coat Na_3_V_2_(PO_4_)_3_ with different B source (BC_3_, B_4_C, BCO_2_, and BC_2_O). It shows that the more BC_2_O and BCO_2_ in the Na_3_V_2_(PO_4_)_3_/carbon composite, the best electrochemical property can be achieved, especially high-rate capability and cyclic stability. This can be attributed to the increased external defects and active sites derived from BC_2_O and BCO_2_ doping, which accelerate the migration of Na^+^ in the carbon layer.

Chen et al. (2018) synthesize F-doping and V-defect Na_3_V_1.98_(PO_4_)_3−x_F_3x_/C composites by solid-state reaction route. F-doping is advanced to short the pathway of Na^+^ diffusion. The Na_3_V_1.98_(PO_4_)_2.9_F_0.3_/C composite delivers an initial charge capacity as high as 143.5 mAh g^−1^ at 0.1 C. After 100 cycles at 1 C, a reversible capacity is 100.6 mAh g^−1^.

Besides, transition metal elements are also commonly used to doping NVP to improve the electrochemical performance. Liu et al. (2019) use Fe element to prepare Fe-doped Na_3_V_2_(PO_4_)_3_@C cathode material. The as-obtained Na_3_V_1.85_Fe_0.15_(PO_4_)_3_@C shows a high capacity of 103.69 mAh g^−1^ and retain a capacity of 94.45 mAh g ^−1^ after 1,200 cycles at 20 C.

### Nanostructure

Reducing particle size of NVP to nanometer scale is another way to shorten Na^+^ diffusion distance, and expand the effective contact area between electrolyte and the active material (Wu et al., 2019). Therefore, cycling performance as well as rate capabilities of Na_3_V_2_(PO_4_)_3_ can be enhanced. However, nanomaterials are easy to agglomerate, leading to an irreversible capacity loss.

**Figure 8:**
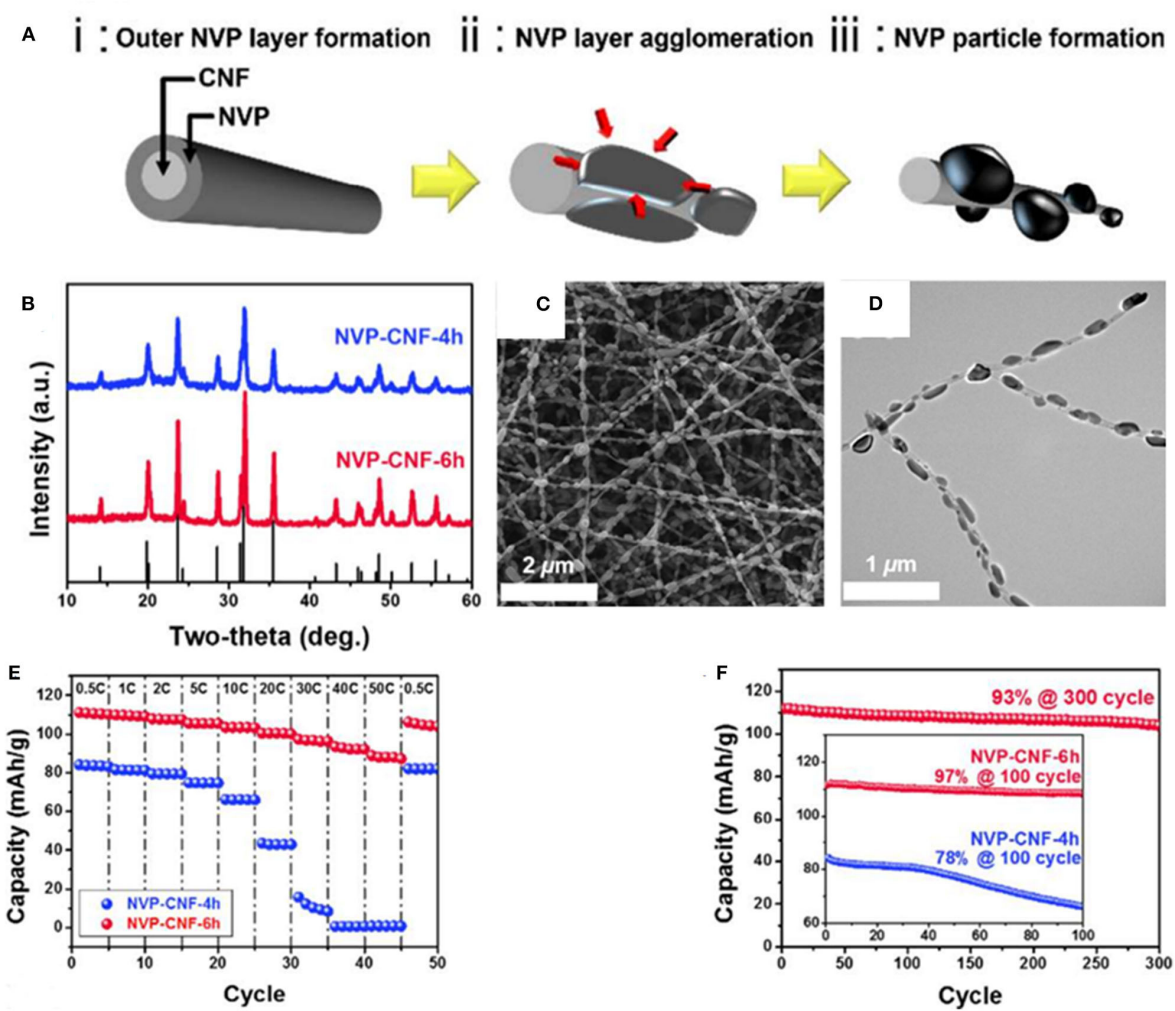
**(A)** The formation process of the NVP-CNF composite by schematic diagram, **(B)** X-ray diffraction patterns of NVP-CNF-4 h and NVP-CNF-6 h, **(C)** SEM image of NVP-CNF composite, **(D)** TEM image of NVP-CNF composite, **(E)** rate property plots of NVP-CNF-4 h and NVP-CNF-6 h, **(F)** cycle property of NVP-CNF-4 h and NVP-CNF-6 h.

As Figure 8A presented, Yang et al. (2015) have embed parts of NVP nanoparticles into carbon nanofibers to obtain Na_3_V_2_(PO_4_)_3_/carbon nanofibers composite (NVP-CNF), which has ultra-high power and excellent cycle performances due to rapid migration of sodium ions along with the conductive CNF. The XRD patterns of the prepared NVP-CNF composites are displayed in Figure 8B. It shows that all the composites have NASICON structure, and belong to the R-3c space group. Figures 8C,D reveal that the NVP nanoparticles have uniformly embedded in the carbon nanofiber. As seen in Figures 8E,F, the NVP-CNF composites have superior rate performances and high capacity retention of ~93% at 1 C after 300 cycles.

**Figure 9:**
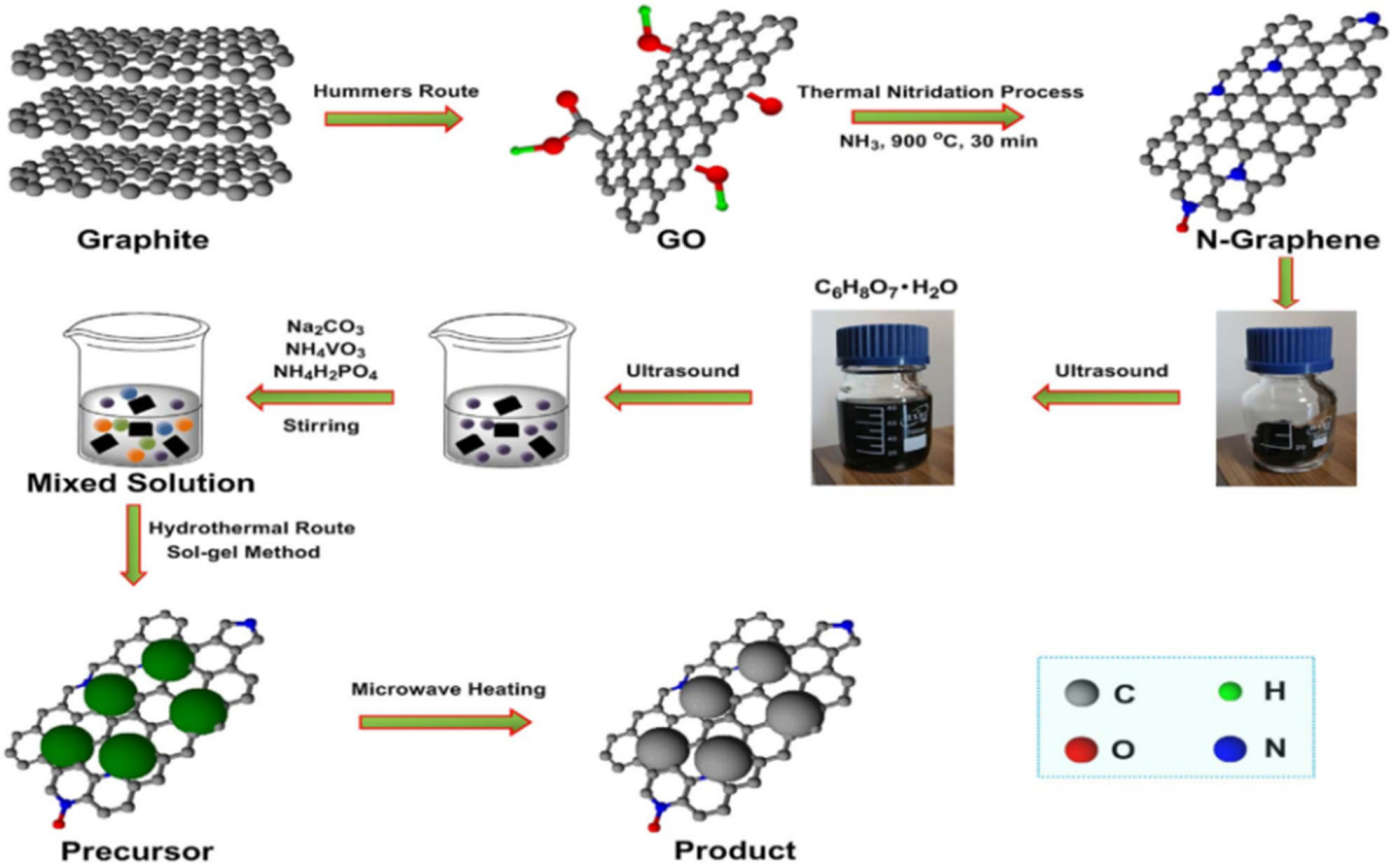
The formation process of the NVP/C@N-graphene composite by schematic diagram.

**Figure 10:**
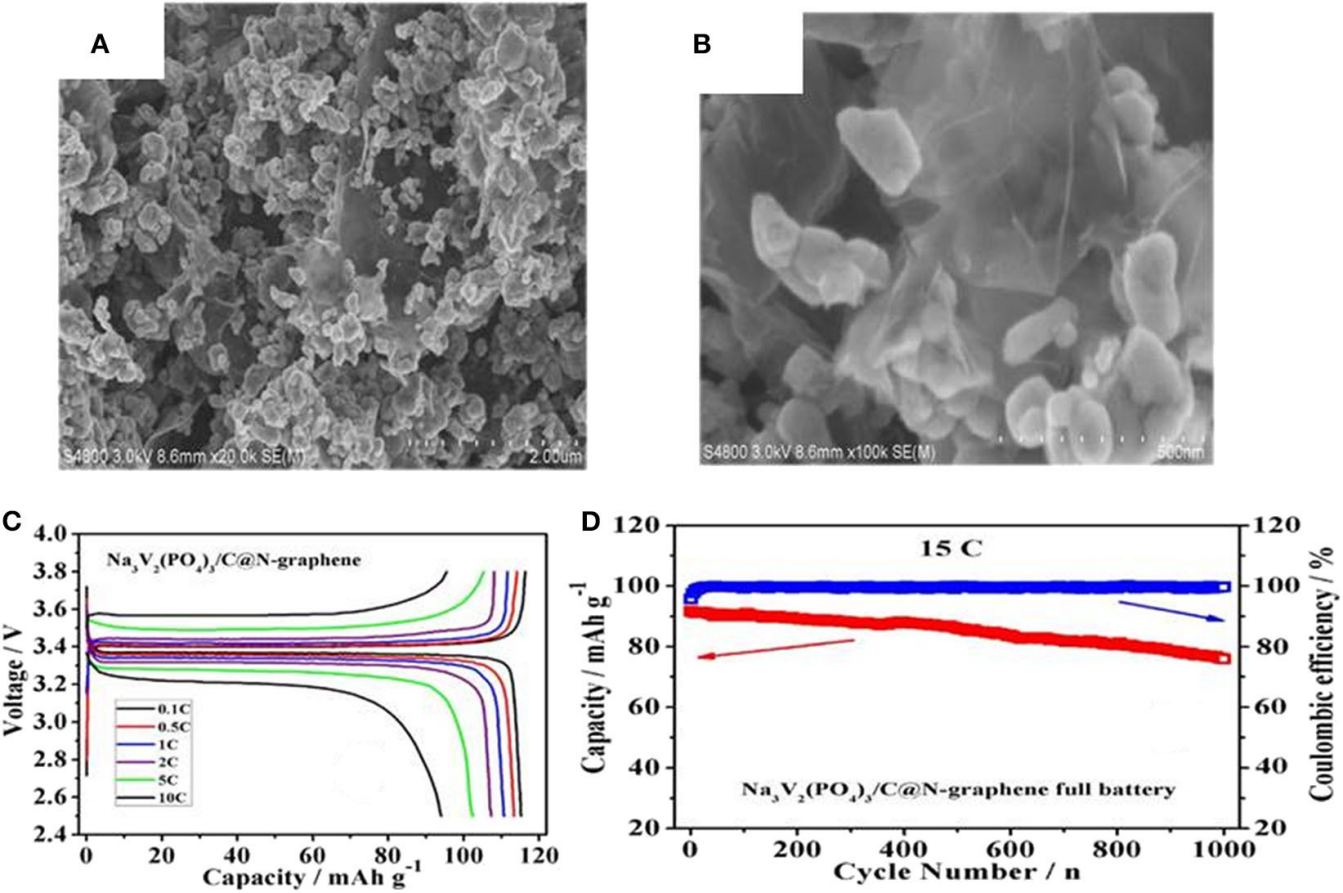
**(A,B)** SEM image of the NVP/C@N-graphene, **(C)** charge-discharge curve of NVP/C@N-graphene, **(D)** cycle property of the NVP/C@N-graphene at 15 C.

As demonstrated in Figure 9, nitrogen doped grapheme (N-graphene) has been used as coating layer to modify Na_3_V_2_(PO_4_)_3_ nanocrystal (NVP/C@N-graphene) (Liu and Guo, 2017). Figures 10A,B display the SEM images of NVP/C@N-graphene, in which the NVP/C particles are anchored on the surface of N-graphene and construct an ideal 3D conductive network. Benefiting from this unique structure, the as-obtained cathode material has an initial specific capacity of 115.2 mAh g^−1^, amounting to about 97.6% of the theoretical capacity of NVP (Figure 10C). In addition, Figure 10D reveals that the composite material still have a high capacity after 1,000 cycles at 15 C, which can be ascribed to the enhanced conductivity by the cross-linked network consisting of carbon-coating layer and N-graphene.

In general, Nano-structure is a promising method to decrease the Na^+^ and electrons diffusion distance inside the NVP cathode materials, thereby improving the electrochemical properties of NVP materials.

### Other Modification Ways

Except for the technologies mentioned above, some other strategies can also enhance cycling stability as well as rate capability of NVP, such as encapsulating NVP into conductive materials (Jung et al., 2013; Tao et al., 2016) or coating it with conductive materials (Hultman et al., 2003; Xiong et al., 2012; Shen et al., 2013, 2016; Song et al., 2013, 2014b,c; Li G. et al., 2014; Si et al., 2014; Guo et al., 2015; Rui et al., 2015; Li et al., 2016, 2017; Xu et al., 2016; Zhou X. S. et al., 2016; Chen L. et al., 2017; Liang et al., 2017; Zhang H. et al., 2017; Gu et al., 2018; Kim et al., 2018; Yi H. M. et al., 2018).

**Figure 11:**
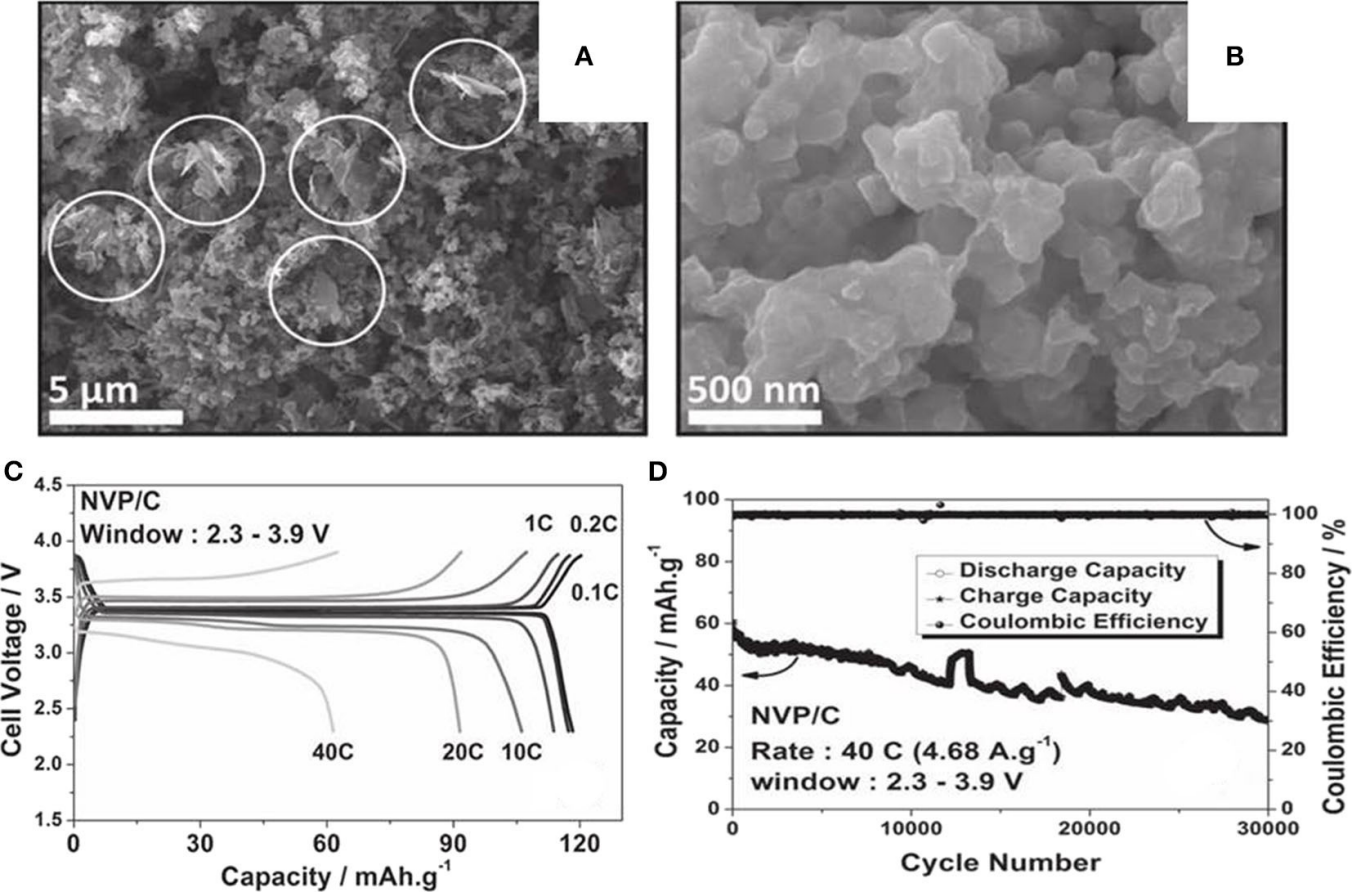
**(A)** FESEM images at low, and **(B)** high magnification of NVP/C, **(C)** galvanostatic cycling of NVP/C at different current rates, **(D)** cycle property of the material cycled at a rate of 40 C for 30,000 cycles.

A porous Na_3_V_2_(PO_4_)_3_/carbon(NVP/C) has been synthesized by a new solution-based method (Saravanan et al., 2013). As we can see from the field emission scanning electron microscope (FESEM) images in Figures 11A,B, NVP/C particles with irregular morphologies and uneven sizes ranging from 500 to 900 nm build up interlinked networks. The composite exhibits initial charge capacity of 50 mAh g^−1^ at 0.2 C, and retains 85% of its primal capacity at 10 C. The first charge-discharge curves of NVP/C at various current rates are shown in Figure 11C, which implies low over potential and good rate behavior. As illustrated in Figure 11D, the composite go through 30,000 cycles and just lose 50% of its original capacity.

**Figure 12:**
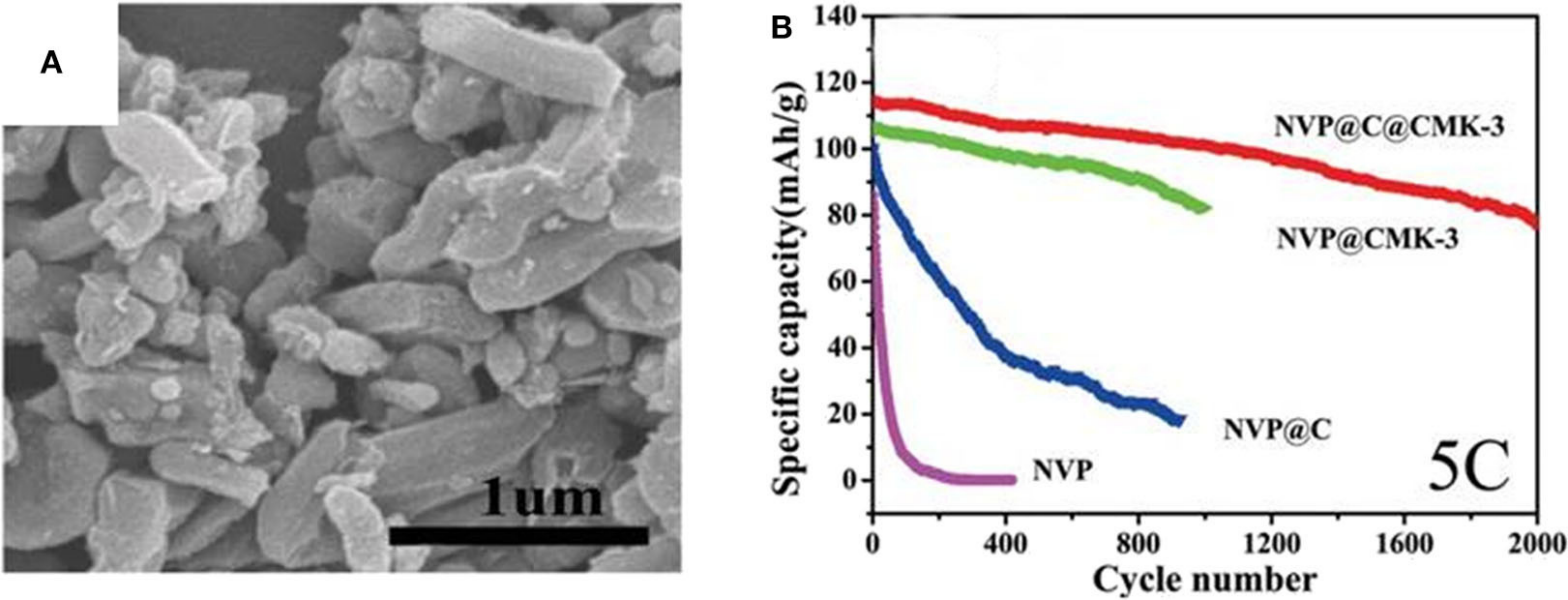
**(A)** SEM image of the NVP@C@CMK-3, **(B)** cycle properties of the NVP,NVP@C, NVP@CMK-3, and NVP@C@CMK-3 at 5 C.

Jiang et al. (2015) have impregnated NVP nanoparticles coated by carbon into a ordered 3D mesoporous interconnected CMK-3(NVP@C@CMK-3). Figure 12A displays that NVP@C@CMK-3 has a rod-like shape, NVP@C particles are encapsulated in the pores of CMK-3. The outer carbon layer could greatly enhance the conductivity of the NVP. As a result, the NVP@C@CMK-3 demonstrates long lifespan over 2,000 cycles and high remaining capacity of 78 mAh g^−1^ at 5 C (Figure 12B), indicating good cycling performances. Compared with bare NVP, faster diffusion of Na^+^ owing to rich porous structure of the NVP@C@CMK-3 improves the cycle capability.

The electrochemical performances of aforementioned NVP cathode materials modified by different methods are compared and listed in Table 2. It is clearly that nanostructured NVP present much better electrochemical performance because they can provide fast Na^+^/electrons migration pathway and more active sites for electrochemical reactions.

**Table 2:** Cycle performances of NVP by various modification methods.

**Modification method**	**Rate/C**	**Cycles**	**Capacity/mAh g^**−1**^**
B doped NVP (Huang et al., 2002)	0.2	40	96.6
Ti doped NVP (Chu and Yue, 2016)	10	200	95.8
Nanostructure (Chen S. Q. et al., 2017)	1	300	104.6
Nanostructure (Lim et al., 2014)	15	1,000	62.7

## Conclusions and Outlook

As one kind of SIBs cathode material, Na_3_V_2_(PO_4_)_3_ has many merits including high energy storage capacities and excellent structural stability. Unfortunately, its large scale applications are impeded by some obstacles. The main challenge of Na_3_V_2_(PO_4_)_3_ is the poor electron conductivity. Besides, as similar as other inserting materials, the volume of Na_3_V_2_(PO_4_)_3_ will change during charge and discharge process. In addition, the crystal structure of Na_3_V_2_(PO_4_)_3_ may change in low temperature. Corresponding expressions have been added in the last part of the revised manuscript.

This review summarizes some common preparing approaches of Na_3_V_2_(PO_4_)_3_ cathode materials and analyzes their effects on the electrochemical of NVP. Sol-gel method is widely used for obtaining electrode materials with homogeneous particle size. Hydrothermal treatment can guarantee high specific surface area and high purity of products. Solid-state method occupies merits of low cost and simple operation process. Electrospinning is suitable to large-scale industrial preparation and able to prepare the uniform materials. NVP cathode materials prepared by all of these methods have various properties, which make the NVP have good prospects for development.

Many kinds of conductive carbon materials are used as coating layer to improve the conductivity of NVP. Various of nanostructured NVP are prepared to shorten the diffusion distance of Na^+^/electrons. Some elements with larger ionic radium are applied to replace Na^+^, for the sake of enlarging volume of NVP and proving more reaction sites. All of these approaches indeed help enhancing the electrochemical performances of Na_3_V_2_(PO_4_)_3_ cathode materials

It is believed that low cost and large-scale material preparation can be achieved by continuous in-depth researches, and Na_3_V_2_(PO_4_)_3_ integrating stable structure, high surface specific are, fast Na^+^/electrons migration channels will be developed. By doing that, the industrial application of high performance NVP cathode materials will be within reach.

## Author Contributions

XZ, JP, and YG contributed the conception and design of the study. XH and HZ organized the database. JP wrote the first draft of the manuscript. XZ and YG revised the whole manuscript.

## Conflict of Interest

HZ was employed by the company Zigong Langxingda Technology Co., Ltd. The remaining authors declare that the research was conducted in the absence of any commercial or financial relationships that could be construed as a potential conflict of interest.
